# Interactions between Canopy Structure and Herbaceous Biomass along Environmental Gradients in Moist Forest and Dry Miombo Woodland of Tanzania

**DOI:** 10.1371/journal.pone.0142784

**Published:** 2015-11-11

**Authors:** Deo D. Shirima, Marion Pfeifer, Philip J. Platts, Ørjan Totland, Stein R. Moe

**Affiliations:** 1 Department of Ecology and Natural Resource Management, Norwegian University of Life Sciences, P.O. Box 5003, 1432 Ås, Norway; 2 Department of Forest Biology, Faculty of Forestry and Nature Conservation, Sokoine University of Agriculture, P.O. Box 3010, Chuo Kikuu, Morogoro, Tanzania; 3 Department of Life Sciences, Imperial College London, Silwood Park Campus, Buckhurst Road, Ascot, Berkshire SL5 7PY, United Kingdom; 4 Department of Biology, University of York, Heslington, York YO10 5DD, United Kingdom; University of Saskatchewan, CANADA

## Abstract

We have limited understanding of how tropical canopy foliage varies along environmental gradients, and how this may in turn affect forest processes and functions. Here, we analyse the relationships between canopy leaf area index (LAI) and above ground herbaceous biomass (AGB_H_) along environmental gradients in a moist forest and miombo woodland in Tanzania. We recorded canopy structure and herbaceous biomass in 100 permanent vegetation plots (20 m × 40 m), stratified by elevation. We quantified tree species richness, evenness, Shannon diversity and predominant height as measures of structural variability, and disturbance (tree stumps), soil nutrients and elevation as indicators of environmental variability. Moist forest and miombo woodland differed substantially with respect to nearly all variables tested. Both structural and environmental variables were found to affect LAI and AGB_H_, the latter being additionally dependent on LAI in moist forest but not in miombo, where other factors are limiting. Combining structural and environmental predictors yielded the most powerful models. In moist forest, they explained 76% and 25% of deviance in LAI and AGB_H_, respectively. In miombo woodland, they explained 82% and 45% of deviance in LAI and AGB_H_. In moist forest, LAI increased non-linearly with predominant height and linearly with tree richness, and decreased with soil nitrogen except under high disturbance. Miombo woodland LAI increased linearly with stem density, soil phosphorous and nitrogen, and decreased linearly with tree species evenness. AGB_H_ in moist forest decreased with LAI at lower elevations whilst increasing slightly at higher elevations. AGB_H_ in miombo woodland increased linearly with soil nitrogen and soil pH. Overall, moist forest plots had denser canopies and lower AGB_H_ compared with miombo plots. Further field studies are encouraged, to disentangle the direct influence of LAI on AGB_H_ from complex interrelationships between stand structure, environmental gradients and disturbance in African forests and woodlands.

## Introduction

Tree species vary in their capacity to use abiotic resources, promoting coexistence among life forms at different growth stages [1,2]. Morphological differences among tree crowns, for example, enhances a forest community’s capacity to exploit light resources and fix carbon, regulating stand-scale biomass production [3]. Canopy leaf area is the main regulator of radiation absorption and can block up to 95% of visible light from reaching the forest floor [4]. Light extinction through dense, multi-layered vegetation creates a strong vertical energy gradient, shaping microclimate and soil properties within the forest [5]. In turn, microclimate, light availability, soil moisture and soil fertility interact to regulate plant growth in sub-canopy layers [6].

Few studies have explored interrelationships between tree diversity, canopy structure and biomass in the Afro-tropics [7,8], and no study has quantified the relationship between canopy structure and herbaceous biomass, or how this varies along gradients of soil nutrients and anthropogenic disturbance. Forests and woodlands in Eastern Africa are under considerable pressure from increasing human populations [9–11]. Land use and climate change interact to modify natural variability in canopy leaf area, which declines with degradation pressure and increases with water availability [12]. The loss of canopy trees due to selective logging and high intensity fires results in forests with simpler vertical structure and reduced functional capacity [13]. This may affect ecosystem productivity, including woody and herbaceous aboveground biomass.

The majority of studies looking at variations in carbon stocks and biodiversity in Africa focus on moist forests [14–17], while miombo woodlands remain understudied [18]. Moist forests tend to have higher biodiversity and carbon value per unit area, but they cover much smaller areas compared with miombo in both Southern and Eastern Africa (0.064 *vs*. 2.7 million km^2^ [9,19]). In Tanzania, miombo woodlands comprise around 90% of the total forested area [20], providing essential resources to rural communities, particularly wood-based energy (firewood and charcoal) and other non-timber products [21,22]. Miombo tree assemblages are predominantly deciduous with open canopies, on soils that have low nutrient content, are well drained, highly leached, acidic and low in organic matter [23]. Moist forests, on the other hand, are predominantly evergreen, with denser canopies on more nutrient-rich soils [24]. Understanding drivers of structure and function in both these vegetation types, especially their role in the carbon cycle, is a priority for research [25].

Here, we analyse the relationships between canopy leaf area index (LAI) and above ground herbaceous biomass (AGB_H_) in a moist forest and miombo woodland in Tanzania. Our objectives are to test the hypotheses that forest structural attributes (tree richness and size distribution) can be used to predict canopy leaf area (LAI), and that AGB_H_ is negatively related to LAI due to light extinction through the canopy. We explore the extent to which our findings vary along environmental and disturbance gradients, and between moist forest and miombo woodland systems.

## Materials and Methods

### Study region

**Fig 1:**
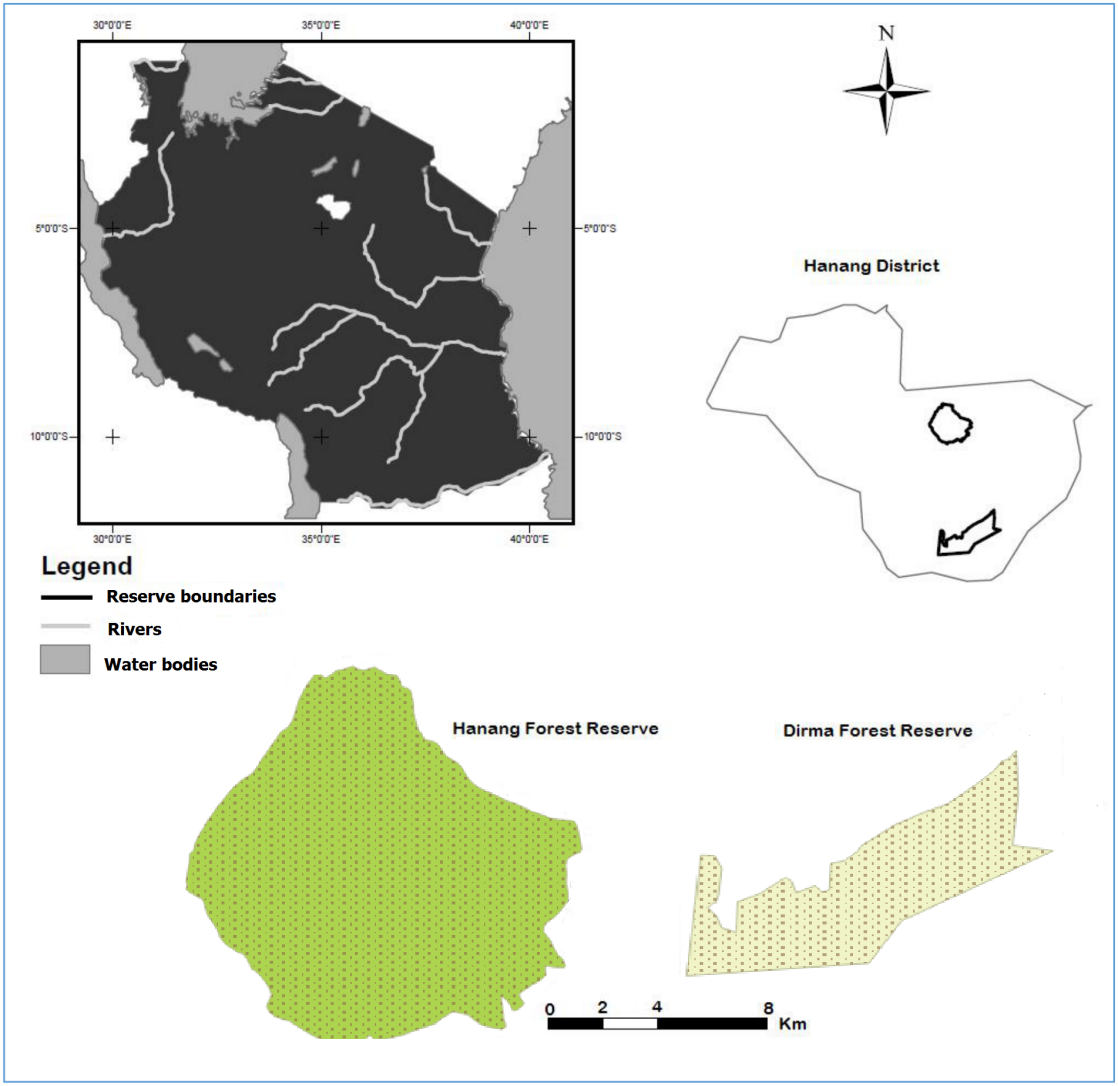
Study area locations in Tanzania.

We conducted our study in Hanang and Dirma forest reserves in Tanzania (Fig 1). Permission to conduct the fieldwork was granted by the Manyara region and Hanang district administrative secretaries. Hanang forest reserve (forest extent: 58.71 km^2^) is a central government catchment reserve, spanning an elevation range of 1860–3418 m (Latitude: -4.44°, Longitude: 35.40°). The reserve receives a mean annual rainfall of 895 mm depending on elevations, ranging from 878 mm at lower elevations to over 1000 mm at higher elevations. Mean annual temperatures range from 17°C at the lowest elevations to 13°C at the highest [26]. Grasses and thickets dominate the highest elevations and steepest slopes. Moist forest with canopy species such as *Albizia gummifera* and *Cassipourea malosana* dominate at mid to high elevations, and are interspaced irregularly by moorland patches. The forest grows on volcanic soils ranging from sandy to humus rich loams in the upland moorlands and upper montane areas [27].

Dirma village forest reserve (miombo extent: 63.5 km^2^, Fig 1) spans an elevation range of 1500–1700 m (Latitude: -4.70°, Longitude: 35.44°). The reserve receives a mean annual rainfall of 796 mm (range: 787–804 mm) and mean annual temperature of 19.5°C (range: 19–20°C) [26]. Miombo woodland assemblages characterized by *Brachystegia spiciformis* and *Julbernadia globiflora* dominate vegetation cover in the reserve. The woodlands grow on poor soils that are low in nutrients and vegetation cover is highly influenced by frequent fires and anthropogenic disturbances, similar to miombo woodlands elsewhere in Africa [9,28].

### Vegetation and soil surveys

We surveyed 100 vegetation plots of 20 m × 40 m (60 plots in moist forest and 40 plots in miombo woodland) in March 2012. The plots were placed systematically along an elevation gradient (from 1583 m and 1900 m minimum elevations in miombo woodland and moist forest respectively), and separated by a minimum distance of 400 m. In each plot, we recorded all tree individuals with diameter at breast height (dbh) ≥ 5 cm, and identified each of these trees to species-level. Where species identification by a botanist was not possible in the field, voucher specimens were collected and identified at Arusha National Herbarium in Tanzania. We recorded geographical location and elevation using a handheld GPS (Map76cx). We measured tree height in the field whenever conditions allowed, using a Suunto-hypsometer. The remaining tree heights were estimated from our field data using biome-specific height-dbh regression equations. We recorded the number of tree stumps in each plot as an indicator of anthropogenic disturbance[29]. Selective logging is widespread in forest and woodland reserves in sub-Saharan Africa, due to acute poverty, increased human populations and weak forest governance [30]. Apart from logging, forest and woodlands in Africa also experience other disturbances such as herbivory and frequent fires.

To estimate AGB_H_, litter biomass and tree seedling density, each plot was sub-divided into eight (10 m × 10 m) subplots. Aboveground herbaceous plant materials (clipped at ground level) and litter materials were collected from five (1 m × 1 m) quadrats, and tree seedlings (dbh < 5cm) were counted in 2 m × 2 m quadrats, placed at random within four alternate subplots (S1 Fig). We recorded the total fresh weight of herbaceous and litter samples in the field. We collected a subset of each of these samples, which were then oven dried in the laboratory to a constant weight at 70°C for 48 h to obtain dry mass estimates. Results were applied to the total fresh weights to obtain the total herbaceous and litter dry mass per plot, referred to as aboveground herbaceous biomass (AGB_H_) and litter biomass.

Soil samples were collected at three depths (0–15 cm, 15–30 cm and 30–60 cm) at five points per plot, i.e. from each of the four corners and from the centre of the main plot. We aggregated samples for each depth into composites for subsequent analyses. The resulting 300 soil samples were air-dried and sieved through a 2 mm wire mesh and analysed for soil pH (at 1:2.5 soil: H_2_O), organic carbon (Walkley-Black method), available phosphorous (Bray II), total nitrogen (Kjeldahl method), potassium and sodium (ammonium acetate 1.0 M pH7.0 extraction) at Seliani Agricultural Research Institute, Arusha, Tanzania.

### LAI data acquisition and processing

All photographs were taken during the wet season in March 2012. Leaf area index was estimated following standard protocols [12,31]. We took 13 hemispherical photographs in each of four subplots (10 m × 10 m) using a Nikon D3100 camera equipped with a hemispherical fish-eye lens (S1 Fig). The camera was mounted on a tripod at 1 m above ground, looking vertically upward from beneath the canopy. The levelled hemispherical photographs were acquired normal to a local horizontal datum, orienting the optical axis of the lens to local zenith. We measured under overcast conditions whenever possible to minimize anisotropy of the sky radiance [32].

CAN-EYE analysis software estimates LAI in digital images based on gap fraction for specific viewing directions. CAN-EYE estimates LAI as plant area index, as is the case with other indirect measurements. Thus, our LAI estimates include materials such as stems, trunks, branches, twigs and plant reproductive parts [33]. However, it is not possible to know if leaves are present behind the stems, branches or trunks. Therefore, masking some parts of the plants to keep only the visible leaves is not correct and could lead to large underestimation of the actual LAI value, depending on the way leaves are grouped in other parts of the plant. Furthermore, during the growing season in both deciduous woodland and evergreen forest, the total vegetation surface is mainly composed of leaf area, and by a lesser part of twigs, branches and stem surface [34]. Also according to [35] branches and boles contributed to total LAI by less than 5% in three relatively dense stands of conifers.

Hemispherical images were pre-processed by first extracting blue-channel pixel brightness values and then applying a threshold algorithm for separating sky from vegetation [36]. Resultant binary images were analyzed using the free canopy analysis software CAN-EYE V6.3.8 [31,37]. For each site, we derived LAI corrected for foliage element clumping [38], limiting the field of view of the lens to values between 0° and 60° to avoid mixed pixels. Values of LAI from the four subplots were averaged per plot for subsequent analyses.

### Forest and woodland structural attributes

Tree richness was estimated as the total number of tree species per plot. Stem and seedling density were estimated as the numbers of tree stems and seedlings per ha. We used Pielous’s index (J) to estimate tree species evenness [39] and the Shannon diversity index (H′) to estimate diversity [40]. We estimated the quadratic mean diameter (QMD) for all trees as $QDM=\sqrt{\left({\bar{d}}^{2}+S^{2}\right)}$, where ${\bar{d}}^{2}$ is the arithmetic mean diameter and *S* ^2^ is the variance of tree diameters in a plot. QMD has a strong correlation to stand volume and basal area, and is a preferred measure of stand structure over the arithmetic mean diameter [41]. We estimated predominant height (PDH) of the forest and woodland stands as the average height of the 100 tallest trees per hectare [42]. Quantified variables were categorised into stand structural and environmental variables for subsequent modelling (Table 1).

**Table 1:** Comparison of stand structural and environmental variables (mean ± SE) measured in moist forest and miombo woodland of Hanang district in Tanzania.

Variables	Forest	Miombo	W*	*P-value*
**Structural attributes**				
Leaf Area Index (LAI)	1.39 ± 0.09	0.93 ± 0.07	2299	0.001
Herbaceous Biomass (Mg ha^-1^)	1.27 ± 0.07	0.91 ± 0.10	1675	0.001
Litter Biomass (Mg ha^-1^)	2.54 ± 0.14	1.82 ± 0.20	1675	0.001
Seedling density ha^-1^	3758 ± 382	3850 ± 222	1055	0.309
Shannon diversity Index	1.54 ± 0.08	1.35 ± 0.07	1500	0.034
Richness	8.85 ± 0.56	6.65 ± 0.43	1567	0.001
Evenness	0.73 ± 0.03	0.74 ± 0.02	1314	0.426
Stem density (trees ha^-1^)	722 ± 56	472 ± 30	1609	0.004
Predominant-Height (PDH; m)	13.66 ± 0.75	9.27 ± 0.49	1789	0.001
Quadratic mean diameter (QMD; cm)	19.54 ± 1.29	14.97 ± 0.66	1497	0.036
**Environmental attributes**				
Elevation (m)	2187 ± 21	1630 ± 30	2400	0.001
Soil pH	5.05 ± 0.01	4.64 ± 0.02	2388	0.001
Soil Organic carbon (%)	1.87 ± 0.08	1.39 ± 0.06	1825	0.001
Soil Phosphorous (mg/Kg)	3.46 ± 0.11	6.40 ± 0.21	45	0.001
Soil Nitrogen (%)	0.19 ± 0.01	0.03 ± 0.00	2398	0.001
Soil Potassium (meq/100g)	1.12 ± 0.33	0.05 ± 0.02	2383	0.001
Soil Sodium (meq/100g)	0.11 ± 0.00	0.06 ± 0.00	2090	0.001
Disturbance	4.26 ± 0.47	3.60 ± 0.43	1271	0.614

* Corresponding Wilcoxon Mann-Whitney test showing the differences in median of the measured parameters between moist forest (N = 60) and miombo woodland (N = 40).

### Modelling vegetation structure and links to environmental drivers

We used generalized linear models (GLM) with Gaussian distribution error and identity link function [43,44] to explore the relationships between stand structural and environmental variables versus LAI. We also explored relationships between stand structural variables (including LAI), and environmental gradients versus AGB_H_. We developed these models for moist forest and miombo woodland, separately.

In a first step, we fitted two subsets of models focussing on: structural variables as predictors of either LAI or AGB_H_; and environmental variables as predictors of either LAI or AGB_H_. Each of these models included disturbance as additional predictor and first term interactions between all predictors. We then combined structural and environmental predictors, disturbance and first term interactions between predictors, into one single model for each response in each vegetation type.

Exploratory analysis using smoother functions [45] indicated nonlinear relationships between LAI, tree richness and predominant height. We therefore fitted relationships including quadratic terms for these predictors (see also S1 Table for details on final global models). We used Pearson correlation (r) and variance inflation factor (VIF) to assess collinearity among structural and environmental predictor variables [46]. In cases of high collinearity between two predictor variables (|r| > 0.5 and VIF > 3.0), we retained the predictor showing a stronger univariate relationship with the response variable [47].

We used stepwise model selection based on the Akaike Information Criterion to identify optimal models from the global models [43,48]. The relative contributions of predictor variables were determined by the percentage reduction in explained deviance (D^2^) [44]. We used likelihood ratio tests to compare subset models with the global model [43]. We validated residual spread and estimated the predictive error using leave-one-out cross-validation [49], implemented using the*“cv*.*glm”* function in R [50], in conjunction with the mean squared error of prediction [51]. Moreover, we used paired Mann-Whitney-Wilcox tests between observed and predicted LAI or AGB_H_ to assess the significance of mean squared error of prediction as a measure of model bias [51].

## Results

### Structural and environmental attributes

We identified 97 tree species from 46 families in moist forest, and 62 species from 29 families in miombo woodland. These two vegetation types differed significantly in their Shannon diversity and tree richness (Table 1). They also differed significantly in LAI, AGB_H_, soil pH, soil phosphorus, soil nitrogen, soil potassium and soil sodium (Table 1).

### Structural and environmental influences on LAI

Structure and environmental variability (combined) explained 76% of the deviance in moist forest LAI and 82% in miombo woodland LAI, outperforming all other models (Table 2). The parameter estimates and the likelihood ratio tests for the combined models were significantly different from zero (P≤0.05), suggesting that these models explained variation in LAI better than the global (unreduced) models for both vegetation types (Table 2 and S2 Table; Forest: AIC = 58.6, LRT_3.6_, P = 0.001, miombo: AIC = 16.8, LRT_17_, P = 0.014). Further, the AIC weights show that the combined models consistently outperform structure-only or environment-only models for LAI, in both moist forest and miombo woodland (Table 2).

**Table 2:** Comparison of alternative models for predicting LAI in moist forest and miombo woodland of Hanang District in Tanzania.

Vegetation type	Predictor sets	Df	D^2^ (%)	AIC	ΔAIC	AIC Weights	LRT	*P-value*	MSEP	W
Forest	Structure	55	64	74.83	16.20	0.00	11.49	0.370	0.20	0.87
	Environment	54	30	116.29	57.70	0.00	9.67	0.210	0.39	0.74
	Combined	51	76	58.60	0.00	0.99	3.60	0.001	0.16	0.98
Woodland	Structure	36	66	22.32	5.50	0.06	6.01	0.400	0.10	0.97
	Environment	35	19	58.72	41.90	0.00	2.32	0.470	0.26	0.89
	Combined	27	82	16.80	0.00	0.94	17.00	0.014	0.09	0.71

Predictor sets include: structural variables only; environmental variables only; structural and environmental variables combined (see Table 1). Results are for models reduced by stepwise selection (see S1 and S2 Tables for details on global models and covariate estimates, respectively). Statistics: D^2^, percent deviance explained; ΔAIC, change in Akaike Information Criterion compared with null model; LRT, likelihood ratio test comparing final models with their respective global models at P ≤ 0.05; MSEP, mean square error of prediction; W, Wilcoxon Mann-Whitney statistic used to estimate prediction bias.

**Fig 2:**
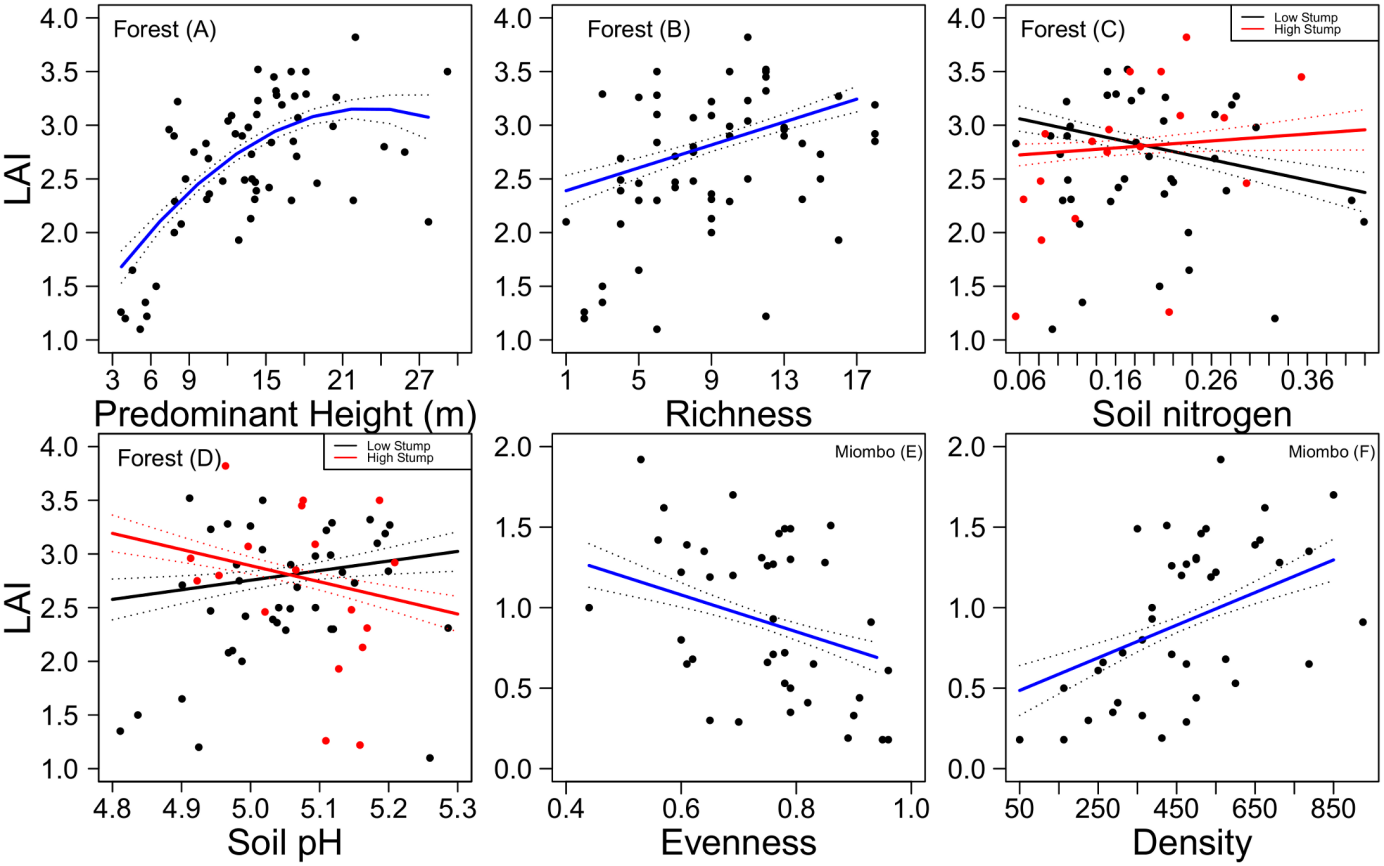
Relationships between leaf area index (LAI), stand structural and environmental variables in moist forest and miombo woodland of Hanang district in Tanzania. (A) LAI shows a non-linear relationship with predominant height, (B) linear relationship with tree species richness, (C) soil nitrogen, (D) soil pH under high disturbance levels, (E) stem density and (F) tree species evenness when all other variables are set to their mean values (S2 Table, combined models). Solid lines plot fitted partial regressions from generalized linear models, with standard errors of the mean in dotted lines.

LAI exhibited a substantial non-linear increase with predominant height and a strong linear increase with tree species richness in moist forest (S2 Table, Fig 2). Additionally, the LAI of moist forest increased weakly with disturbance, but decreased with soil nitrogen (see S2 Table, S2 Fig). Under high disturbance, LAI of moist forest increased with soil nitrogen and decreased with soil pH (S2 Table, Fig 2).

**Fig 3:**
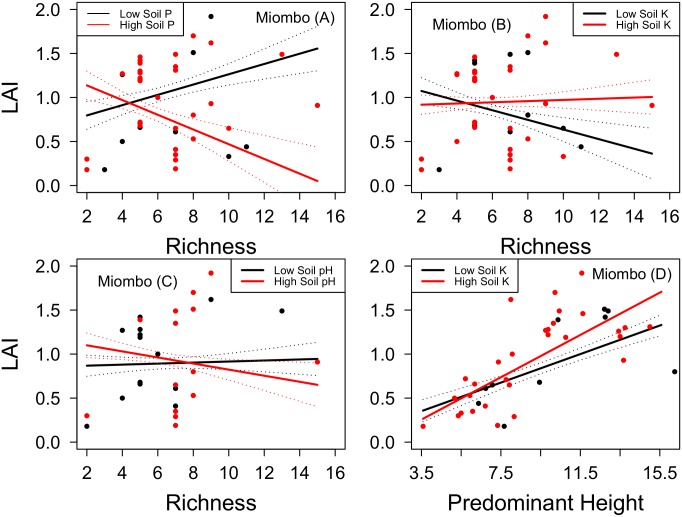
Relationships between leaf area index (LAI) and, stand structural and environmental variables in miombo woodland of Hanang district in Tanzania. (A) LAI shows linear relationships with tree species richness at high soil phosphorous, (B) with tree species richness at high soil potassium, (C) with tree species richness at high soil pH, and (D) with predominant height at low and high soil potassium, when all other variables are set to their mean values (S2 Table, combined model). The solid lines are the fitted partial regression lines from generalized linear models of the relationships between LAI and labeled variables (Low and High levels of P = phosphorous, K = potassium and pH, respectively), with standard errors of the mean in dotted lines.

In miombo woodland, LAI decreased linearly with tree species evenness and increased strongly with stem density (S2 Table, Fig 2). The LAI of miombo woodland increased with soil phosphorous and nitrogen (S2 Table). Moreover, tree species richness interacted with soil phosphorous and pH in that LAI decreased with richness in soils rich in phosphorous and at high pH while LAI increased with richness where soil phosphorous concentrations and pH were low (S2 Table, Fig 3). Plant species richness was negatively related to LAI when soil potassium was low but at high soil potassium, the relationship between richness and LAI was positive (S2 Table, Fig 3). There was an overall positive relationship between predominant height and LAI, and the relationship was stronger at relatively high soil potassium (S2 Table, Fig 3).

### Structural and environmental influences on AGB_H_

Structural and environmental variability (combined) explained around 25% and 45% of deviance in AGB_H_ of moist forest and miombo woodland, respectively. The parameter estimates and the likelihood ratio tests for these two models were significantly different from zero (P ≤ 0.05), suggesting that they explained deviance in AGB_H_ better than the global model in miombo woodland (Table 3 and S3 Table; miombo: AIC = 46.2, LRT_69.38_, P = 0.001), while in moist forest only the parameter estimates were significant (P ≤ 0.05). The AIC weights shows that the combined models are the most optimal models for predicting AGB_H_ in miombo woodland and moist forest (Table 3).

**Table 3:** Comparison of alternative models predicting aboveground herbaceous biomass (AGB_H_) in moist forest and miombo woodland of Hanang District in Tanzania.

Vegetation type	Predictor sets	Df	D^2^ (%)	AIC	ΔAIC	AIC Weights	LRT	*P-value*	MSEP	W
Forest	Structure	56	19	129.05	0.58	0.43	8.34	0.400	0.48	0.00
	Environment	56	5.5	138.20	9.73	0.00	5.63	0.340	0.61	0.00
	Combined	54	25	128.47	0.00	0.57	51.50	0.150	0.47	0.00
Woodland	Structure	36	26	54.14	7.94	0.02	18.60	0.080	0.21	0.97
	Environment	35	27	55.60	9.40	0.00	4.95	0.210	0.26	0.87
	Combined	31	45	46.20	0.00	0.97	69.38	0.001	0.17	0.81

Predictor sets and statistics as in Table 2. Results are for models reduced by stepwise selection (see S1 and S2 Tables for details on global models and covariate estimates, respectively).

**Fig 4:**
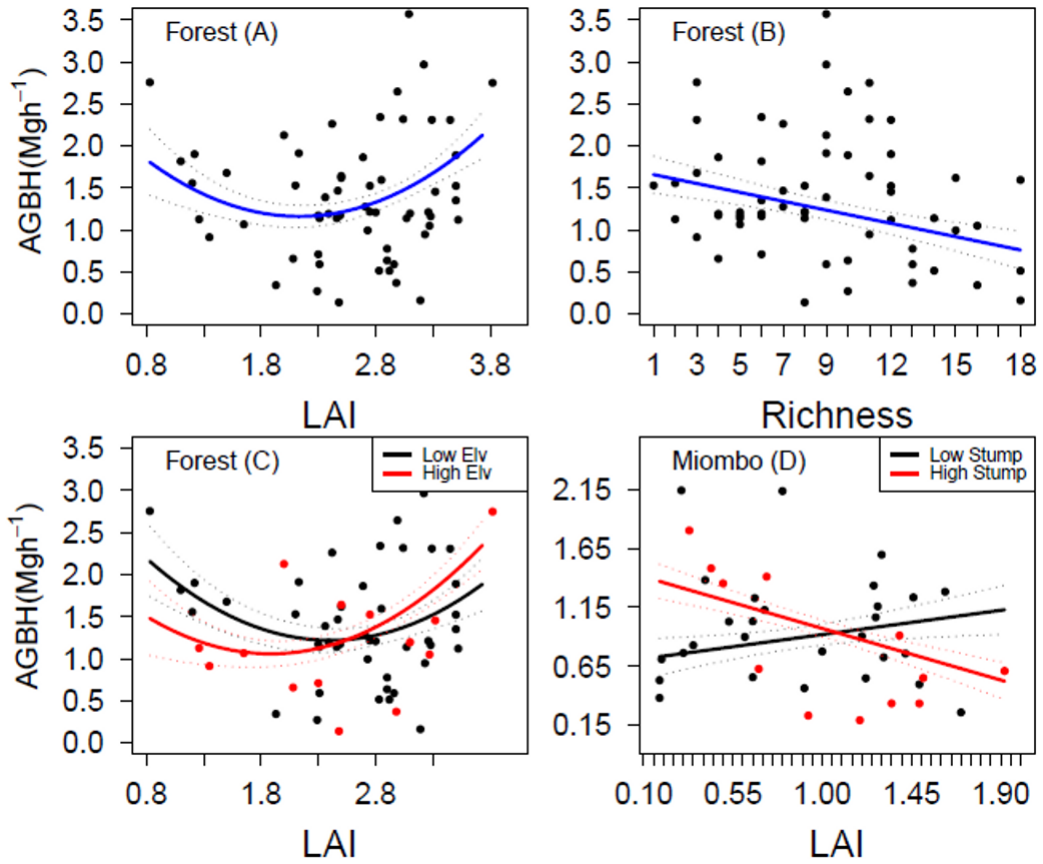
Relationships between aboveground herbaceous biomass (AGB_H_), stand structural and environmental variables in moist forest and miombo woodland of Hanang district in Tanzania. (A) AGB_H_ show a non-linear relationship with leaf area index, (B) linear relationship with tree species richness, (C) a non-linear relationship with LAI at high elevation in moist forest, and (D) linear relationship with LAI in miombo woodland at high levels of disturbance, when all other variables are set to their mean values (S3 Table, combined models). The solid lines are the fitted partial regression lines from generalized linear models of the relationships between AGB_H_ and labeled variables (Low-Elv, High-Elv, and Low-Stump and High-Stump are low and high levels of elevation and disturbance gradients, respectively), with standard errors of the mean in dotted lines.

AGB_H_ in moist forest decreased linearly with tree species richness, but followed an inverted hump-shape curve in response to LAI (S3 Table, Fig 4). AGB_H_ in moist forest decreased with LAI at lower elevations whilst increasing with LAI at higher elevations (S3 Table, Fig 4). AGB_H_ in miombo woodland increased linearly with soil nitrogen and soil pH (S2 Fig), but decreased linearly with LAI at high levels of disturbance (S3 Table, Fig 4).

## Discussion

### Structural and environmental influences on LAI

Foliage density and tree sizes affect the ability of woody vegetation types to intercept light and atmospheric nutrients [3]. Thus, differences in canopy leaf area are likely to feed through to plant growth and biomass production and the vegetation type’s carbon sequestration potential. We found substantial differences in structural and environmental variables between the moist forest and miombo woodland, which in turn correlated with differences in LAI and herbaceous biomass.

Structural and environmental variables (combined) explained over 70% of the deviance in LAI and performed better (AIC weights of over 90%) than either structural or environmental variables alone_,_ in both vegetation types, which suggests that forests and woodlands respond to the environment based on morphological and physiological adaptations [52]. We have shown that LAI increases with tree richness in moist forest and additionally with tree stem density in miombo woodland, similar to findings in tropical lowland and montane forests in Ecuador [53]. Our data indicated that tree richness and stem density had low correlation (about 50%), suggesting that the observed patterns of LAI vs richness and LAI vs stem density in miombo woodland were not artifacts of one-another. This finding lends support to the hypothesis of resource use complementary and higher productivity in more diverse forests [53]. Whereas in less diverse and less productive miombo woodland, LAI decreased with tree species evenness, suggesting that a few dominant species with relatively low multi-layer canopy which characterize the canopy leaf area.

We show that while LAI increases with tree height, this relationship is not linear and LAI saturates or decreases for forest stands featuring larger trees, a pattern we find for moist forest only. Similar findings have been reported for Acadia forest in the US [54], where the gradual decline in LAI with tree height was attributed to severe branch abrasion and loss of new foliage as trees grow taller [39]. Yet, our finding contradicts a global meta-analysis, which reports positive, non-saturating relationships between remotely-sensed LAI and field-measured tree height across broadleaf forests and savanna [55]. The difference in findings may partly arise from the different spatial scales and resolutions used in Yuan et al.’s [55] study, and uncertainty introduced by different instruments used to estimate LAI at different sites in this meta-analysis. However, it should also be noted that LAI estimated from hemispherical images saturates in high-biomass biomes due to methodological constraints such as inability to differentiate leaves from other parts of the plant such as trunks, branches or twigs [32].

We show that anthropogenic disturbance and/or its interaction with soil nutrients influenced variability in LAI of moist forest. In miombo woodland, anthropogenic disturbance had less influence on LAI compared to soil nutrients or interactions between soil nutrients and structural variables. Miombo woodlands are relatively highly disturbed (selective logging, frequent fires grazing and shifting cultivation), have more open canopies and are more limited by soil nutrients [23], compared to moist forest. Therefore, disturbance inferred from stump frequency will likely correspond to a more pronounced effect on LAI in moist forest than in miombo woodland. Further studies are encouraged to disentangle anthropogenic effects from those of soil and other structural attributes on the moist forest and miombo woodland canopy characteristics.

### Structural and environmental influences on AGB_H_

As the herbaceous layer is likely to affect soil, surface fluxes, and ground-dwelling organisms, understanding how canopy structure interacts with this layer can aid in understanding the responses of sub-canopy biodiversity and ecosystem processes to climate and land use [31,53]. Previous studies suggested that trees can benefit herbaceous vegetation through amelioration of harsh environmental conditions and increased nutrient availability [6,56]. However, trees have also been reported to suppress herbaceous biomass by altering light availability and soil fertility on the forest floors [57].

In miombo woodland, structural and environmental variability explained 45% of the deviance in AGB_H_, and performed better (AIC weight of over 90%) than either structural or environmental gradients alone. In moist forest, structural and environmental variability performed better than structural variables (AIC weights, 57% vs 43%) but by smaller margin than in miombo woodland. AGB_H_ in the moist forest decreased with increasing LAI, which increased with tree species richness. This suggests that tree richness may suppress AGB_H_ by increasing canopy density thereby reducing light availability at the forest floor limiting herbaceous plant growth. Patterns are complex, though, as indicated by the U-shaped association between AGB_H_ and LAI. The slight increase in AGB_H_ with LAI at high elevations suggests a reduced impact of light limitation due to declines in tree growth at higher altitudes [58]. This is indicative of a positive effect of environmental stress gradients (adverse climate, shallow soils and low radiant energy) on plant growth at high elevations, which promotes plant coexistence [59] due to low competition intensity [60–62].

In miombo woodland, AGB_H_ was higher under denser vegetation canopies, suggesting an amelioration of harsher environments characterized by either drought or frequent fires. Miombo features open canopies, and sub-canopy plant growth is likely to be soil nutrient and water-limited rather than restricted by light availability. Unsurprisingly, disturbance, which further prevents canopy closure, interacts with soil nutrients and LAI to regulate biomass in the herbaceous layer. Trees’ multiple effects on the herbaceous layer partly depend on interactions between tree canopy (i.e. shedding), soil fertility and moisture availability [63]. Soil nutrients from decaying tree stumps or ring-backed roots and deposits from biomass burning have been associated with increased herbaceous biomass in miombo woodland [23,64]. Meanwhile, human activities may affect AGB_H_ depending on disturbance type (i.e. grazing and fire). For example, herbaceous biomass decreased linearly with LAI at high disturbance levels and increased linearly with soil nitrogen and pH, indicating high local variation in herbaceous plant growth in response to stand structure characteristics and soil nutrients in miombo woodlands.

## Conclusions

Tree diversity, sizes and environmental variability affect canopy leaf area and herbaceous biomass in both moist forest and miombo woodland. Our findings provide support for the hypothesis of niche complementarity, with higher tree diversity enabling a better use of canopy space optimizing light capture through forest canopies. Since canopy leaf area is a good indicator of a vegetation’s photosynthetic capacity, it is likely that high species richness in moist forests facilitates higher productivity and biomass production. Denser moist forest canopies, on the other hand, should have negative impact on herbaceous biomass than less dense canopy miombo woodlands [56]. Our data provide support for this hypothesis, but suggest complex interrelationships between environmental and structural parameters interacting to drive variability in herbaceous biomass. Anthropogenic disturbance modifies canopy structure and herbaceous biomass, and also affects abiotic parameters including soil nutrients [65]. Monitoring of structural components (i.e. tree species diversity, sizes, forms and LAI), anthropogenic disturbances and their interactions with environmental factors is important for effective management of human-modified moist forest and miombo woodland ecosystems in Tanzania, and elsewhere in Africa.

## Supporting Information

S1 Fig A sketch diagram of plot design used in the moist forest and miombo woodland of Hanang district in Tanzania. (TIFF)

S2 Fig Relationships between LAI or AGB_H_, and structural, environmental variables, and their combination in moist forest and miombo woodland. (Forest 1) LAI show linear relationships with soil nitrogen in moist forest, (Miombo 2) with soil nitrogen, (Miombo 3) soil phosphorous, and (Miombo 4) soil potassium in Miombo woodland. (Forest 5) AGB_H_ show linear relationships with elevation in moist forest, (Miombo 6) with soil nitrogen, (Miombo 7) soil pH and (Miombo 8) with disturbance in miombo woodland when all other variables are set to their mean values. The solid lines are the fitted partial regression lines with 95% shaded confidence band.
(TIFF)

S1 Table Global models used in predicting LAI and AGB_H_ in moist forest and miombo woodland of Hanang district in Tanzania. Models used either structural, environmental variables, or a combination of both. Note: Only predicator variables with variance inflation factor (VIF) ≤ 3 and Pearson correlation coefficient (r) ≤ 50% were included in the model.
(DOCX)

S2 Table Results of generalized linear models predicting LAI as a function of structural variables, environmental variables, and their combination in moist forest and miombo woodland of Hanang district in Tanzania. (DOCX)

S3 Table Results of generalized linear models predicting aboveground herbaceous biomass (AGB_H_) as function of structural variables and environmental variables, and their combinations in moist forest and miombo woodland of Hanang district in Tanzania. (DOCX)
