# Supplementary figures and images for: Interactions between Canopy Structure and Herbaceous Biomass along Environmental Gradients in Moist Forest and Dry Miombo Woodland of Tanzania
Source: PLoS One. 2015 Nov 11;10(11):e0142784. doi: 10.1371/journal.pone.0142784 (PMC4641655; doi:10.1371/journal.pone.0142784)

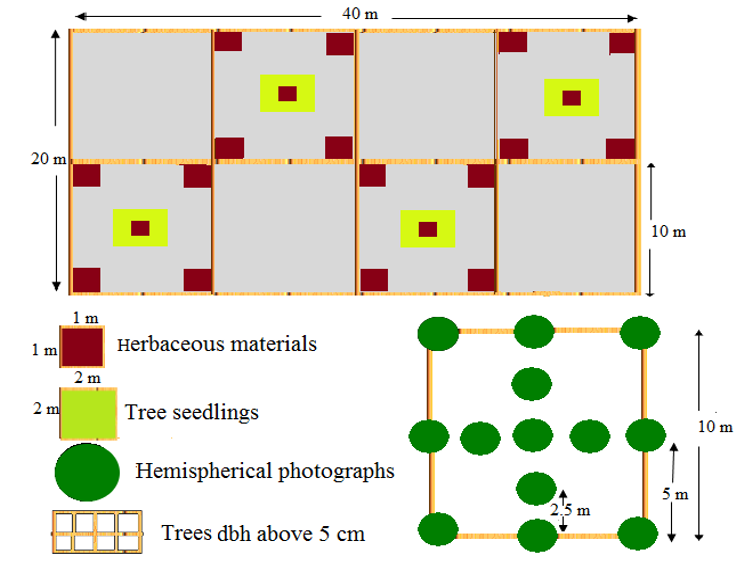

Supplement: S1 Fig — (TIFF) [file pone.0142784.s001.tiff]

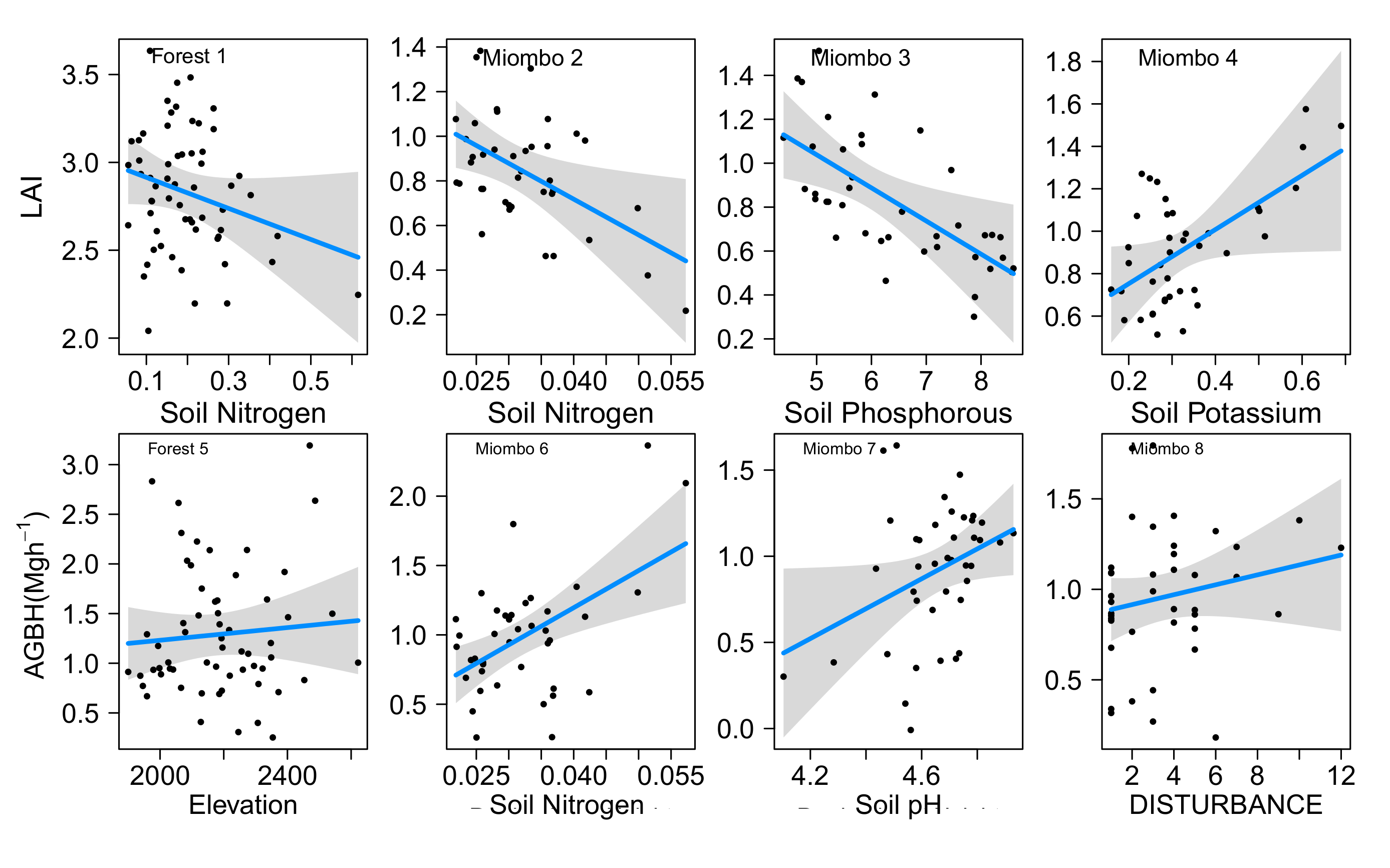

Supplement: S2 Fig — (Forest 1) LAI show linear relationships with soil nitrogen in moist forest, (Miombo 2) with soil nitrogen, (Miombo 3) soil phosphorous, and (Miombo 4) soil potassium in Miombo woodland. (Forest 5) AGBH show linear relationships with elevation in moist forest, (Miombo 6) with soil nitrogen, (Miombo 7) soil pH and (Miombo 8) with disturbance in miombo woodland when all other variables are set to their mean values. The solid lines are the fitted partial regression lines with 95% shaded confidence band. (TIFF) [file pone.0142784.s002.tiff]
