# Supplementary material for: Interactions between Canopy Structure and Herbaceous Biomass along Environmental Gradients in Moist Forest and Dry Miombo Woodland of Tanzania
Source: PLoS One. 2015 Nov 11;10(11):e0142784. doi: 10.1371/journal.pone.0142784 (PMC4641655; doi:10.1371/journal.pone.0142784)
Supplement: S1 Table — Models used either structural, environmental variables, or a combination of both. Note: Only predicator variables with variance inflation factor (VIF) ≤ 3 and Pearson correlation coefficient (r) ≤ 50% were included in the model. (DOCX) [file pone.0142784.s003.docx]

**S1 Table:** Global models used in predicting LAI and AGB_H_ in moist forest and miombo woodland of Hanang district in Tanzania. Models used either structural, environmental variables, or a combination of both. Note: Only predicator variables with variance inflation factor (VIF) ≤ 3 and Pearson correlation coefficient (r) ≤ 50% were included in the model.

| Model | Model parameters | Description |
| --- | --- | --- |
| 1 | LAI = Richness+ Richness^2^ + Evenness + Stem density + Predominant height+ predominant height^2^ + disturbance | The relationships between LAI, stand structural attributes and disturbance |
| 2 | LAI = Phosphorus + Potassium + Nitrogen + pH + Elevation + disturbance | The relationships between LAI, environmental gradients and disturbance |
| 3 | LAI = Richness + Richness^2^ + Evenness + Stem density + Predominant height + predominant height^2^ + Phosphorus + Potassium + Nitrogen + pH + Elevation + disturbance | The relationships between LAI, stand structural attributes, environmental gradients and disturbance |
| 1 | AGB_H_ = Richness + Evenness + Stem density + Quadratic mean diameter + predominant height + LAI + LAI^2^ + Seedlings + Litter biomass + disturbance | The relationships between AGB_H,_ stand structural attributes, LAI and_,_ disturbance |
| 2 | AGB_H_ =Phosphorus + Potassium + Nitrogen + pH + Elevation + disturbance | The relationships between AGB_H_, environmental gradients disturbance |
| 3 | AGB_H_ = Richness + Evenness+ Stem density + Quadratic mean diameter + Predominant height + LAI + LAI^2^ + Seedlings + Litter biomass + Phosphorus + Potassium + Nitrogen + pH + Elevation + disturbance | The relationships between AGB_H_ stand structural attributes, environmental gradients and disturbance |
