# Supplementary material for: Interactions between Canopy Structure and Herbaceous Biomass along Environmental Gradients in Moist Forest and Dry Miombo Woodland of Tanzania
Source: PLoS One. 2015 Nov 11;10(11):e0142784. doi: 10.1371/journal.pone.0142784 (PMC4641655; doi:10.1371/journal.pone.0142784)
Supplement: S2 Table — (DOCX) [file pone.0142784.s004.docx]

**S2 Table.** Results of generalized linear models predicting LAI as a function of structural variables, environmental variables, and their combination in moist forest and miombo woodland of Hanang district in Tanzania.

| **Models** | **Final model** | **Estimates** | **D^2^ (%)** | **AIC** | **Df** | **LRT** | ***P*** | **MSEP** | **W** |
| --- | --- | --- | --- | --- | --- | --- | --- | --- | --- |
| **Forest** |  |  |  |  |  |  |  |  |  |
| Structure | Slope: ( *p [t]=* 0.001) | 0.04 | 64.00 | 74.83 | 1 | 11.49 | 0.370 | 0.20 | 0.87 |
|  | Richness | 0.17 | 18.00 |  |  |  | 0.003 |  |  |
|  | (Richness)^2^ | -0.01 | 11.00 |  |  |  | 0.015 |  |  |
|  | Predominant-Height | 0.19 | 42.00 |  |  |  | 0.001 |  |  |
|  | (Predominant-Height)^2^ | -0.00 | 14.00 |  |  |  | 0.004 |  |  |
| Environment | Slope: ( *p [t]=* 0.001) | -16.58 | 30.00 | 116.29 | 1 | 9.67 | 0.210 | 0.39 | 0.74 |
|  | Disturbance | 3.49 | 0.50 |  |  |  | 0.013 |  |  |
|  | Soil Nitrogen | -2.14 | 0.30 |  |  |  | 0.110 |  |  |
|  | Soil pH | 3.86 | 3.00 |  |  |  | 0.003 |  |  |
|  | Disturbance: Soil Nitrogen | 0.65 | 18.00 |  |  |  | 0.030 |  |  |
|  | Disturbance: Soil pH | -0.71 | 11.00 |  |  |  | 0.010 |  |  |
| Combined | Slope: ( *p [t]=* 0.001) | -5.88 | 76.00 | 58.60 | 1 | 3.60 | 0.001 | 0.16 | 0.98 |
|  | Richness | 0.05 | 0.00 |  |  |  | 0.001 |  |  |
|  | Predominant-Height | 0.18 | 3.00 |  |  |  | 0.001 |  |  |
|  | (Predominant-Height)^2^ | 0.00 | 0.00 |  |  |  | 0.002 |  |  |
|  | Disturbance | 2.32 | 19.00 |  |  |  | 0.008 |  |  |
|  | Soil Nitrogen | -2.42 | 42.00 |  |  |  | 0.006 |  |  |
|  | Soil pH | 1.37 | 17.00 |  |  |  | 0.090 |  |  |
|  | Disturbance: Soil Nitrogen | 0.51 | 25.00 |  |  |  | 0.005 |  |  |
|  | Disturbance: Soil pH | -0.48 | 14.00 |  |  |  | 0.005 |  |  |
| **Woodland** |  |  |  |  |  |  |  |  |  |
| Structure | Slope: ( *p [t]=* 0.001) | -1.01 | 66.00 | 22.32 | 1 | 6.01 | 0.400 | 0.10 | 0.97 |
|  | Density | 0.13 | 31.00 |  |  |  | 0.030 |  |  |
|  | Predominant-Height | 0.34 | 44.00 |  |  |  | 0.010 |  |  |
|  | (Predominant-Height)^2^ | -0.01 | 11.00 |  |  |  | 0.040 |  |  |
| Environment | Slope: ( *p [t]=* 0.04) | -16.58 | 19.00 | 58.72 | 1 | 2.32 | 0.470 | 0.26 | 0.89 |
|  | Disturbance | 3.49 | 2.80 |  |  |  | 0.170 |  |  |
|  | Soil potassium | -2.14 | 1.10 |  |  |  | 0.050 |  |  |
|  | Soil Nitrogen | 3.86 | 0.70 |  |  |  | 0.120 |  |  |
|  | Soil pH | 0.65 | 15.00 |  |  |  | 0.020 |  |  |
| Combined | Slope: ( *p [t]=* 0.001) | -0.68 | 82.00 | 16.80 | 1 | 17.0 | 0.014 | 0.09 | 0.71 |
|  | Richness | 0.26 | 2.00 |  |  |  | 0.067 |  |  |
|  | Evenness | -6.14 | 25.00 |  |  |  | 0.014 |  |  |
|  | Density | -16.04 | 18.00 |  |  |  | 0.024 |  |  |
|  | Predominant-Height | 1.77 | 50.00 |  |  |  | 0.082 |  |  |
|  | Soil Phosphorous | 1.23 | 1.00 |  |  |  | 0.022 |  |  |
|  | Soil potassium | -1.14 | 0.00 |  |  |  | 0.011 |  |  |
|  | Soil Nitrogen | 0.19 | 4.00 |  |  |  | 0.006 |  |  |
|  | Soil pH | -0.01 | 1.00 |  |  |  | 0.756 |  |  |
|  | Phosphorous : Richness | -0.06 | 5.00 |  |  |  | 0.012 |  |  |
|  | Potassium : Richness | 0.59 | 4.00 |  |  |  | 0.018 |  |  |
|  | Potassium : Predominant-Height | 0.37 | 14.00 |  |  |  | 0.013 |  |  |
|  | Soil pH :Richness | -0.23 | 15.00 |  |  |  | 0.039 |  |  |

Optimal model terms, their percent deviance explained (D^2^; in italics for each predictor term and bolded for the entire reduced model), their comparable full models using likelihood ratio test (LRT) at α = 0.05), probability deviation from a slope of zero (p[t]), mean square error of prediction (MSEP), Akaike Information Criterion (AIC), and Wilcoxon Mann-Whitney test (W) prediction bias test. See S1Table for details on global models.
