# Supplementary material for: Interactions between Canopy Structure and Herbaceous Biomass along Environmental Gradients in Moist Forest and Dry Miombo Woodland of Tanzania
Source: PLoS One. 2015 Nov 11;10(11):e0142784. doi: 10.1371/journal.pone.0142784 (PMC4641655; doi:10.1371/journal.pone.0142784)
Supplement: S3 Table — (DOCX) [file pone.0142784.s005.docx]

**S3 Table.** Results of generalized linear models predicting aboveground herbaceous biomass (AGB_H_) as function of structure variables and environmental variables, and their combinations in moist forest and miombo woodland of Hanang district in Tanzania.

| **Models** | **Final model** | **Estimates** | **D^2^ (%)** | **AIC** | **Df** | **LRT** | ***P*** | **MSEP** | **W** |
| --- | --- | --- | --- | --- | --- | --- | --- | --- | --- |
| **Forest** |  |  |  |  |  |  |  |  |  |
| Structure | Slope: ( *p [t]*= 0.052) | 4.55 | 19.00 | 129.05 | 1 | 8.34 | 0.400 | 0.48 | 0.001 |
|  | Litter biomass | -0.35 | 3.00 |  |  |  | 0.060 |  |  |
|  | LAI | -2.51 | 0.00 |  |  |  | 0.001 |  |  |
|  | (LAI)^2^ | 0.53 | 17.00 |  |  |  | 0.001 |  |  |
| Environment | Slope: ( *p [t]*= 0.61) | 1.46 | 5.50 | 138.20 | 1 | 5.63 | 0.340 | 0.61 | 0.001 |
|  | Elevation | -0.18 | 0.00 |  |  |  | 0.240 |  |  |
|  | Disturbance | -0.02 | 1.00 |  |  |  | 0.540 |  |  |
|  | Elevation : Disturbance | 0.05 | 5.00 |  |  |  | 0.010 |  |  |
| Combined | Slope: ( *p [t]*= 0.052) | -6.52 | 25.00 | 128.47 | 1 | 51.50 | 0.150 | 0.47 | 0.001 |
|  | Elevation | 0.26 | 0.00 |  |  |  | 0.050 |  |  |
|  | Richness | -6.14 | 5.00 |  |  |  | 0.020 |  |  |
|  | LAI | -16.04 | 3.00 |  |  |  | 0.005 |  |  |
|  | (LAI)^2^ | 1.77 | 12.00 |  |  |  | 0.020 |  |  |
|  | Elevation : LAI | 1.23 | 7.00 |  |  |  | 0.050 |  |  |
| **Woodland** |  |  |  |  |  |  |  |  |  |
| Structure | Slope: ( *p [t]*= 0.022) | 0.66 | 26.00 | 54.14 | 1 | 18.60 | 0.080 | 0.21 | 0.97 |
|  | disturbance | 0.17 | 0.00 |  |  |  | 0.001 |  |  |
|  | LAI | 0.25 | 8.00 |  |  |  | 0.290 |  |  |
|  | Disturbance : LAI | -0.16 | 19.00 |  |  |  | 0.001 |  |  |
| Environment | Slope: ( *p [t]*= 0.021) | 2.33 | 27.00 | 55.60 | 1 | 4.95 | 0.210 | 0.26 | 0.87 |
|  | Nitrogen | 27.59 | 13.00 |  |  |  | 0.001 |  |  |
|  | Soil pH | -0.49 | 7.00 |  |  |  | 0.560 |  |  |
|  | Disturbance | -1.95 | 0.00 |  |  |  | 0.070 |  |  |
|  | Soil pH : disturbance | 0.42 | 9.00 |  |  |  | 0.070 |  |  |
| Combined | Slope: ( *p [t]*= 0.001) | -4.40 | 45.00 | 46.20 | 1 | 69.38 | 0.001 | 0.17 | 0.81 |
|  | Soil Nitrogen | 26.77 | 13.00 |  |  |  | 0.001 |  |  |
|  | Soil pH | 0.87 | 7.00 |  |  |  | 0.050 |  |  |
|  | Disturbance | 0.19 | 0.00 |  |  |  | 0.001 |  |  |
|  | LAI | 0.41 | 5.00 |  |  |  | 0.070 |  |  |
|  | Disturbance : LAI | -0.18 | 28.00 |  |  |  | 0.001 |  |  |

Optimal model terms, their percent deviance explained (D2; in italics for each predictor term and bolded for the entire reduced model), their comparable full models using likelihood ratio test (LRT) at α = 0.05), probability deviation from a slope of zero (p[t]), mean square error of prediction (MSEP), Akaike Information Criterion (AIC), and Wilcoxon Mann-Whitney test (W) prediction bias test. See S1Table for details on global models.
